# Altered functional brain networks in problematic smartphone and social media use: resting-state fMRI study

**DOI:** 10.1007/s11682-023-00825-y

**Published:** 2023-12-05

**Authors:** Eszter Áfra, József Janszky, Gábor Perlaki, Gergely Orsi, Szilvia Anett Nagy, Ákos Arató, Anna Szente, Husamalddin Ali Mohammad Alhour, Gréta Kis-Jakab, Gergely Darnai

**Affiliations:** 1 Department of Behavioral Sciences, Medical School, University of Pécs, Pécs, Hungary; 2 Department of Neurology, Medical School, University of Pécs, Pécs, Hungary; 3 HUN-REN-PTE Clinical Neuroscience MR Research Group, Pécs, Hungary; 4 Department of Neurology, University of Pécs, Pécs, Hungary; 5 Pécs Diagnostic Centre, Pécs, Hungary; 6 Department of Neurosurgery, Medical School, University of Pécs, Pécs, Hungary; 7 Neurobiology of Stress Research Group, Szentágothai Research Centre, University of Pécs, Pécs, Hungary; 8 Department of Laboratory Medicine, Medical School, University of Pécs, Pécs, Hungary

**Keywords:** Functional magnetic resonance imaging, Resting-state, Functional brain networks, Language network, Visual network

## Abstract

Nowadays, the limitless availability to the World Wide Web can lead to general Internet misuse and dependence. Currently, smartphone and social media use belong to the most prevalent Internet-related behavioral addiction forms. However, the neurobiological background of these Internet-related behavioral addictions is not sufficiently explored. In this study, these addiction forms were assessed with self-reported questionnaires. Resting-state functional magnetic resonance imaging was acquired for all participants (*n* = 59, 29 males) to examine functional brain networks. The resting-state networks that were discovered using independent component analysis were analyzed to estimate within network differences. Significant negative associations with social media addiction and smartphone addiction were found in the language network, the lateral visual networks, the auditory network, the sensorimotor network, the executive network and the frontoparietal network. These results suggest that problematic smartphone and social media use are associated with sensory processing and higher cognitive functioning.

## Introduction

Currently, the fastest growing behavioral problem is problematic Internet use (PIU) which has gained worldwide attention (Dieter et al., 2017) and it has become a serious public health threat (Sevelko et al., 2018). The clinical features of PIU are similar to those of substance use disorders, including uncontrolled use, obsessive thinking about the Internet and neglecting everyday life (Csibi et al., 2018). However, Internet use-related disorders, except internet gaming disorder (IGD), are not yet mentioned in the Diagnostic and Statistical Manual of Mental Disorders (DSM-5; American Psychiatric Association).

Social networking sites (SNS) enable users to socialize with friends and access information about others (Park & Kim, 2013). Overuse of social networking services might lead to a specific form of PIU, which is defined as a maladaptive dependency on SNS (He et al., 2017). Excessive SNS use is often associated with excessive smartphone use, since smartphones have become the most popular way to access the Internet and social networks (Lee et al., 2021). Activities offered by smartphones might lead to a myriad of negative consequences. Several physical and psychological outcomes have been reported, including mental fatigue, low self-esteem and a tendency toward depressive or dysphoric states (Wilmer et al., 2017).

Internet-related addictions are relatively new terms and their neurobiological mechanisms are still not completely understood. Resting-state functional connectivity (rs-FC) analysis allows the identification of neural circuitry dysfunctions in several neuropsychiatric conditions, including addictions. Rs-FC circuit-alterations are less likely to be disconcerted by subtle task-based paradigms, the identified networks are consistent across time between and within individuals and they reflect the cognitive elements for task-processing (Sutherland et al., 2012). Independent component analysis (ICA) is a reliable and commonly used method for the evaluation of resting-state data. In ICA, the complex data can be decomposed into independent subparts (called components), revealing the consistent resting-state networks (RSNs) of the brain (Pariyadath et al., 2016). These RSNs are in accordance with the known architecture of the functional systems related to sensory, motor and cognitive functions (Wang et al., 2017) and proved to play a role in human behavior (He et al., 2017).

Previous resting-state studies showed that the default mode network (DMN)—which is engaged when awake individuals are not performing any specific tasks—is altered in PIU (Wang et al., 2017, 2019). Further RS studies found altered visual attentional network (Wang et al., 2019) in problematic Internet users. Impaired frontoparietal network (FPN) was also found in PIU (Hong et al., 2013; Wang et al., 2017), which is related to a wide range of cognitive tasks. The salience network (SN) is involved in orienting attention toward relevant stimuli (Pariyadath et al., 2016). It was found to be altered in PIU as well. Attentional malfunctions in the main two attentional networks were found in subjects with social network and smartphone addiction (Wang et al., 2019). RS fMRI studies focusing on smartphone overuse also confirmed the involvement of some of these networks. Ahn et al. (2021) found enhanced functional connectivity between the salience and DMN in problematic smartphone users. Static functional connectivity within the frontoparietal network was also showed to be strengthened in problematic smartphone users as revealed by Liu and colleagues (Liu et al., 2022, for review see Montag & Becker, 2023).

In this study, we investigated whether smartphone addiction and social network addiction – that are specific forms of PIU—are associated with functional brain alterations in RSNs. In order to explore the full pattern of brain functional connections, we used ICA then examined the within-network connectivity with dual regression and the between-network connectivity with FSLNets. We think that by using a whole brain approach instead of using a small number of pre-identified seed-regions (as has been conducted in previous research), we can provide a more complete picture of the underlying neural mechanisms of Internet-related addictions. Compared to previous studies here we investigate young adults (some previous studies focused on adolescents), we assess SNS and smartphone overuse (previous RS studies focused merely on PIU and online gaming) and as described before we used a whole brain approach instead of using a pre-defined regions-of-interest or networks.

## Methods

### Participants

Participants were recruited through an online questionnaire. From the 523 applicants who completed the questionnaire, 72 (36 males) were randomly chosen*.* All participants were healthy and aged between 18 and 30 (mean ± SD: 24.69 ± 3.23) years*.* All participants underwent a brief interview via telephone to screen out individuals with neurological problems. Subjects with chronic illnesses, neurological or psychiatric disorders were not included. According to the Edinburgh Handedness Inventory (Oldfield, 1971), all participants had right-hand dominance (range 0.40–1.00). One participant with excessive head motion during MRI was excluded from the study. Nine subjects showed symptoms of moderate or severe depression according to the Beck Depression Inventory (BDI) (Beck et al., 1961) and a radiologist expert found three subjects with minor anatomical anomalies, they were excluded from further analyses. Therefore, the final group included 59 (29 males) participants. Each person was paid a small fee at the end of the measurements for the participation. The study was approved by the National Medical Research Council (registration number: 6843-5/2021/EÜIG). All procedures performed in this study were in accordance with the ethical standards of the institutional and national research committee and with the 1964 Declaration of Helsinki and its later amendments or comparable ethical standards. A written informed consent was obtained from all participants in the study.

### Questionnaires

Since there are no widely accepted diagnostic criteria or cutoff points for internet-related addictions it is highly recommended to measure excessive internet use with questionnaires and use scores as continuous variables (Poli, 2017).

Bergen Social Media Addiction Scale (BSMAS) which was validated by Bányai et al. (2017). This tool identifies the signs of problematic social media use. The Hungarian version contains 6 items answered on a 5-point scale (never to always). These items were developed to refer to the previously mentioned six core criteria of addictions (salience, tolerance, mood modification, withdrawal symptoms, conflict, and relapse).

Smartphone Application-Based Addiction Scale (SABAS) (Csibi et al., 2018) was also used, which is a self-reported questionnaire, created to screen the risks of smartphone application-based addiction. It contains six items that refer to the core criteria of addictions. The items are rated using a 6-point Likert-type scale ranging from 1 (strongly disagree) to 6 (strongly agree). Higher scores in SABAS indicate a greater risk of developing addiction to smartphone application use.

### Procedure

In the scanner, the participants were instructed to rest and keep their eyes open for 10 min, while focusing on the white fixation cross overlaid on black background. Participants were asked to stay awake and try not to think about anything. The paradigm was implemented using the Presentation software (Version 17.1, Neurobehavioral Systems Inc., Berkeley, CA, USA) and the stimuli were presented using an MRI-compatible LCD screen (BOLDscreen 24 LCD for fMRI, Cambridge Research Systems Ltd., Rochester, United Kingdom).

### MRI data acquisition

All measurements were performed on a 3 T MAGNETOM Prisma^fit^, human whole-body MRI scanner (Siemens AG, Erlangen, Germany) with a 20-channel head/neck coil.

Functional images were acquired using a standard 2D echo planar imaging (EPI) sequence with the following parameters: TR/TE: 3000/30 ms; flip angle (FA): 83°; field of view (FOV): 210 × 210 mm^2^; 70 × 70 matrix; 44 axial slices with a thickness of 3 mm; 2040 Hz/pixel receiver bandwidth, no gap; interleaved slice order to avoid crosstalk between continuous slices.

For distortion correction purposes, field mapping sequence was used (TR/TE1/TE2 = 480/4.92/7.38 ms; Flip angle = 60°; 44 axial slices; FOV = 210 × 210 mm^2^; matrix size = 70 × 70; receiver bandwidth = 290 Hz/pixel) with identical voxel size.

Anatomical images for registration purposes were obtained using a T1-weighted axial 3D-MPRAGE sequence (TR/TE/TI: 2530/3.4/1100 ms; FA: 7°; FOV: 256 × 256 mm^2^; 256 × 256 matrix; slice thickness: 1 mm; 176 slices, 200 Hz/pixel receiver bandwidth).

### fMRI data pre-processing

Pre-processing of resting state fMRI data was carried out by using FMRIB’s Software Library (FSL v6.0; http://www.fmrib.ox.ac.uk/fsl).

Pre-processing included MCFLIRT motion correction, slice timing correction, brain extraction, spatial smoothing with 5 mm full width at half maximum, EPI distortion correction with FSL FUGUE, FSL FIX (https://fsl.fmrib.ox.ac.uk/fsl/fslwiki/FIX/UserGuide) and a high-pass temporal filter of 100 s. The single-session data sets were registered into the MNI152 standard space using a two-step process. First, the functional (EPI) image of each subject was registered to that subject’s T1 structural scan using BBR (6 degrees-of-freedom). Then, each subject’s T1 image was registered to the 2 mm MNI152 standard space T1 image using a 12 degrees-of-freedom linear fit followed by nonlinear registration (FNIRT, warp resolution = 10 mm). Next, for each subject, these two registrations were combined and applied to the first-level statistical maps to take them into standard space.

### Within network analysis

To identify RSNs, the preprocessed functional data were temporally concatenated in order to create a single data set using ICA in FSL MELODIC. This concatenated data was decomposed into 25 independent components (ICs) to create a study-specific average of RSNs. This number of dimensionalities is in line with some previous studies which were able to reliably explore RSNs (Damoiseaux et al., 2006; Nomi & Uddin, 2015; Smith et al., 2014). Moreover, small dimensionalities typically provide an estimate of whole resting-state networks compared to high dimensionalities, which are used to define smaller nodes. Six components were determined to be noise-related artefacts representing motion, physiology and scanner-related effects (components 20–25). The remaining 19 ICs of interest were used in further analysis. After visual inspection, we also conducted spatial correlation between the 19 ICs with well-known RSN templates obtained by Smith et al. (2009) using the fslcc command from FSL.

To examine within-network differences in FC, the *dual regression* approach in FSL (v6.0) was applied. This approach allows us the voxel-wise comparisons of RS functional connectivity. In the first stage, the group-average set of spatial maps was regressed for each subject separately into the subject's 4D space-time dataset. This resulted in a set of subject-specific timeseries, one per group-level IC. In the second stage, subject-specific timeseries are regressed into the same 4D dataset, resulting in a set of subject-specific spatial maps, one per group-level IC. Non-parametric permutation testing with 5000 permutations was used to create correlation maps of questionnaire scores. The resulting correlation maps were thresholded using threshold-free cluster enhancement with an alpha level of 0.05 (family wise error – FWE – corrected). Depression scores (BDI) and anxiety scores (Trait Anxiety Inventory) were used as covariates in the regression models. Because of the high intercorrelation between the questionnaire scores (as shown in Table 1.), SABAS and BSMAS scores were first evaluated separately then the two variables were used in a common statistical model to explore independent effects.

**Table 1:** Descriptives of the self-reported questionnaires

Scores	Median	Min	Max	BSMAS	SABAS
BSMAS	**9**	**6**	**22**	1.00	
SABAS	**19**	**10**	**32**	r = 0.64	1.00

*BSMAS* bergen social media addiction scale, *SABAS* smartphone application-based addiction scale

### Between-network analysis

Between-network connectivity was examined with FSLNets v0.6 analysis package implemented in MATLAB. This method involved taking each subject’s timecourses from the dual regression to subject them to between-network comparisons. The aim of this method was to determine how ICs are correlated with each other (Smith et al., 2014), that results in a 19 × 19 correlation matrix. To get a better estimate of the direct connections between nodes, partial correlation coefficients (with rho = 0.1 in Ridge Regression option) were used that were converted from Pearson’s r-values into z-statistics with Fisher's transformation. Questionnaire scores were then used to predict partial correlation values using FSL randomize with 5000 permutations (FWE corrected). Depression scores and anxiety scores were used as covariates.

## Results

**Fig. 1:**
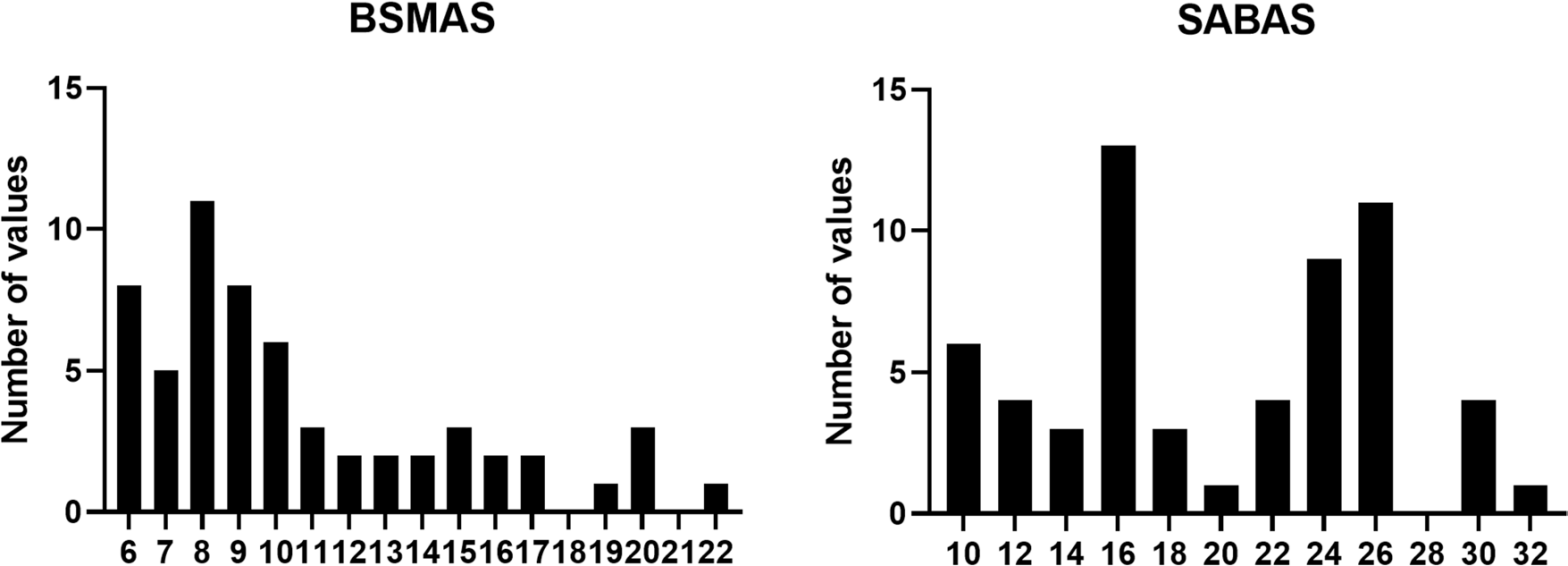
Histograms showing the distribution of the questionnaires’ data. BSMAS: Bergen Social Media Addiction Scale; SABAS: Smartphone Application-Based Addiction Scale

Descriptive results of the self-reported questionnaires’ data and the correlation coefficient between them are summarized in Table 1, while the distributions of the questionnaire scores are shown in Fig. 1.

### Dual regression group ICA

**Fig. 2.**
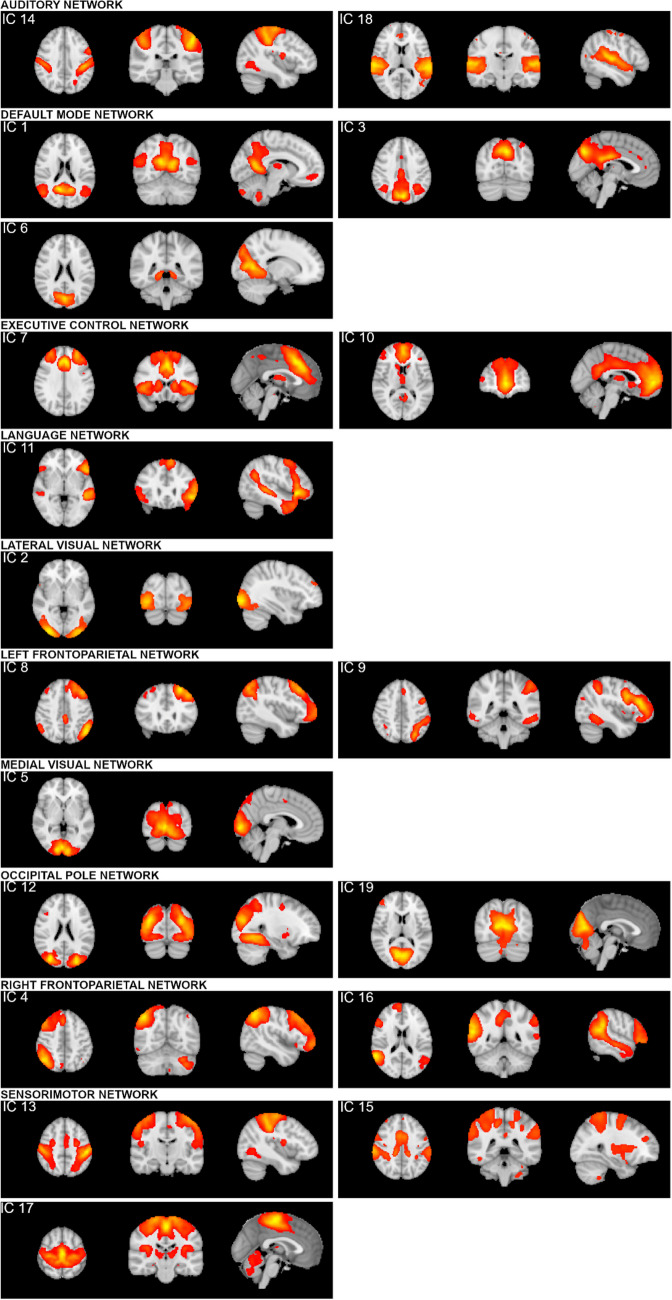
19 components revealed by the independent component analysis. Auditory network: IC 14, IC 18; Default mode network: IC 1, IC 3, IC 6; Executive control network: IC 7, IC 10; Language network: IC 11; Lateral visual network: IC 2; Left frontoparietal network: IC 8, IC 9; Medial visual network: IC 5; Occipital pole: IC 12, IC 19; Right frontoparietal network: IC 4, IC 16; Sensorimotor network: IC 13, IC 15, IC 17. Red-yellow color bars depict Z scores ranging from 3.0 to 32.27

According to the templates by Smith et al. (2009), we revealed three visual components (the medial visual network, occipital pole and lateral visual network), the DMN, sensorimotor network, auditory network, executive control network and the left and the right frontoparietal networks (the spatial correlation coefficient was higher than 0.3 in all cases) (Fig. 2).

Cerebellar RSN did not appear in this study due to the incomplete coverage of the cerebellum during the scanning. Two components (IC 6 and IC 11) could not be identified according to the Smith’s templates (the spatial correlation coefficient was lower than 0.3); therefore, we used functional network templates made by Stanford's Functional Imaging in the neuropsychiatric disorders lab (Shirer et al., 2012) (http://findlab.stanford.edu/functionalROIs.html) to identify these components. Accordingly, IC 6 represents a component that includes voxels of the (ventral) default mode network and IC 11 includes voxels of the language network.

### Within-network correlations with the questionnaire scores

**Fig. 3:**
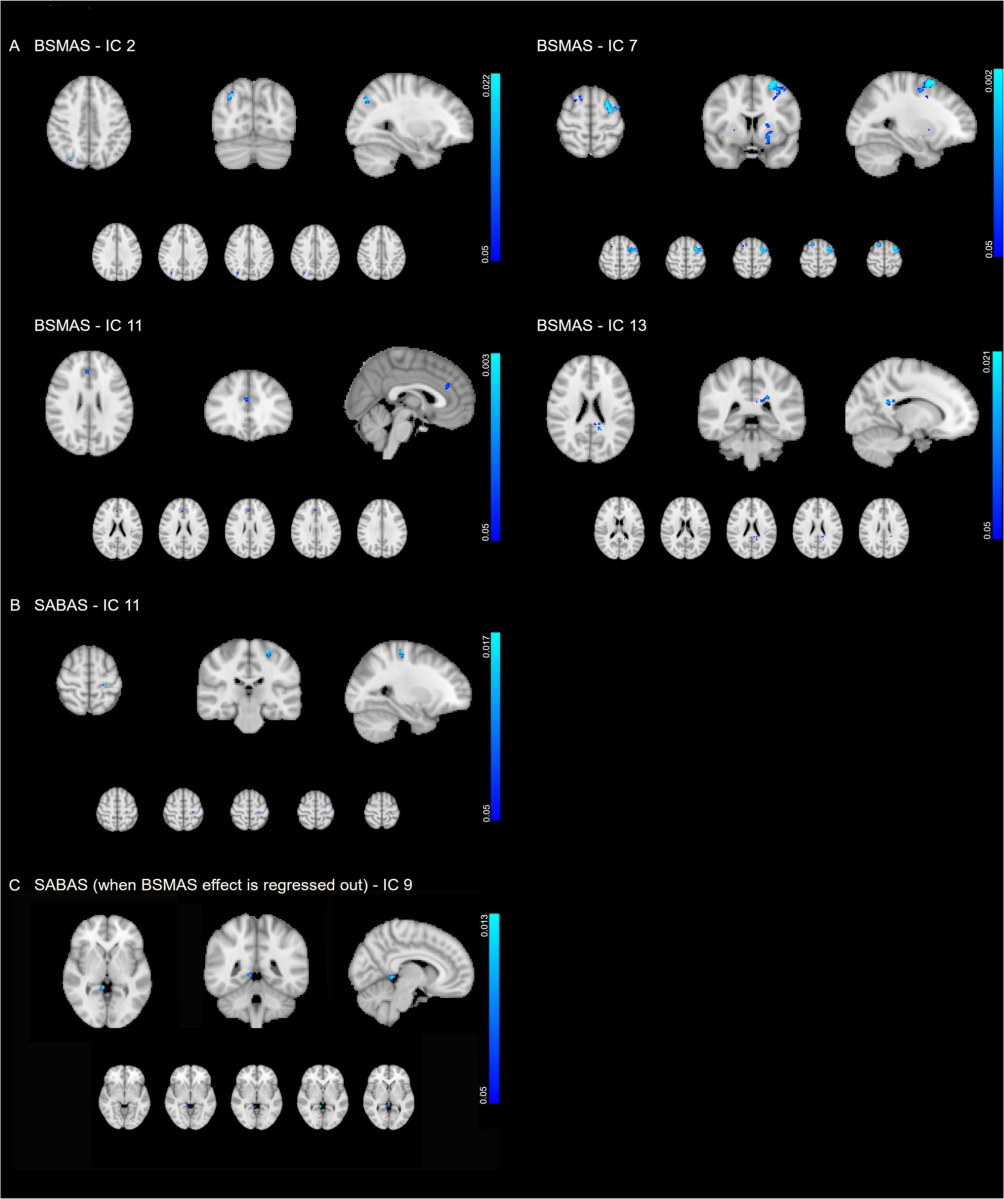
Group-level negative associations between **A**) BSMAS and FC within the IC 2 (lateral visual network), IC 7 (executive control network), IC 11 (language network), IC 13 (sensorimotor network; **B**) SABAS and FC within the IC 11 (language network) and **C**) SABAS (when BSMAS effect is regressed out and FC within the IC 9 (left frontoparietal network). SABAS: Smartphone Application-Based Addiction Scale; BSMAS: Bergen Social Media Addiction Scale; Blue-light blue color bars depict FWE corrected *P*-values. Axial slices are shown in radiological convention

Four components showed negative correlations with BSMAS. These components contain voxels of the language network (IC 11), sensorimotor network (IC 13), bilateral executive control network (IC 7) and lateral visual network (IC 2). FC in IC 11 showed negative association with the SABAS scale. This IC represents a network that includes voxels of the language network (Table 2, Fig. 3). In the common statistical model SABAS also showed negative independent effect on the left frontoparietal network (IC 9). No other significant result was revealed.

**Table 2:** Independent components showing negative associations with questionnaire scores. FWE corrected (for multiple comparisons across voxels) *P*-values are reported

Component	Cluster	Area	Voxels	Min p-value	MNI coordinates		
					X	Y	Z
BSMAS							
IC 2		**Lateral visual network**					
	C1	Right lateral occipital cortex	33	0.022	32	−72	36
IC 7		**Executive control network**					
	C1	Left middle frontal gyrus	462	0.002	−26	4	60
		Left superior frontal gyrus					
		Left precentral gyrus					
	C2	Right superior frontal gyrus	119	0.005	16	16	64
	C3	Right insular cortex	91	0.035	18	18	−2
		Right frontal operculum cortex					
	C4	Right frontal orbital cortex	49	0.03	42	26	−10
	C5	Right insular cortex	26	0.029	36	18	−10
		Right frontal orbital cortex					
IC 11		**Language network**					
	C1	Anterior cingulate gyrus	28	0.027	4	32	24
		Right paracingulate gyrus					
IC 13		Sensorimotor network					
	C1	Posterior cingulate cortex	21	0.028	−20	−38	28
SABAS							
IC 11		**Language network**					
	C1	Left precentral gyrus	28	0.017	−24	−28	62
		Left postcentral gyrus					
SABAS (when BSMAS effect regressed out)							
IC 9		**Left frontoparietal network**					
	C1	Lingual gyrus	30	0.013	8	−42	0
		Posterior cingulate cortex					

*BSMAS* bergen social media addiction scale, *SABAS* smartphone application-based addiction scale

### Between-network connectivity

No significant result was revealed.

## Discussion

In this study, we investigated whether smartphone and social network addictions are related to FC alterations, within and between the well-known RSNs. We found some significant associations that will be discussed separately.

The lateral visual network and the sensorimotor network were found to be linked to social media addiction. These findings are in line with previous studies investigating IGD in the sense that increased visual stimulation alters the visual systems. Studies focusing on IGD revealed enhanced FC within the visual networks and between visual areas and other RSNs, such as auditory, somatosensory and visuospatial networks (Dong et al., 2012; Wang et al., 2016; Zheng et al., 2019). The authors of these articles speculate that the long-term online-gaming experience may increase FC in the visual networks and enhance player's coordination skills among visual, sensorimotor, auditory and visuospatial systems. Unfortunately, studies of other Internet-related addiction forms are sparse and found indirect, non-decisive evidence; e.g., Wang et al. (2019) found decreased visual attentional network functioning, which underlies associations between PIU and visual attention deficits, however the primary cause behind these deficits may be the attention deficit per se and not visual network alterations. Moreover, Horvath et al. (2020) found that smartphone addiction has a negative impact on daily functioning through brain areas linked to visual and auditory processing. However, the link between the daily malfunctioning and the smartphone addiction is again ambiguous. To the best of our knowledge, this is the first study to show direct evidence that problematic social media use is inversely associated with the FC in a visual network. It is important to point out that, unlike IGD, the relationship here is clearly negative. This may be due to the visual stimulation and passive use of the devices compared to computer gamers. However, with the lack of behavioral evidence, this explanation must be considered speculative.

Our data-driven approach showed that decreased FC in the language network is associated with higher SABAS and higher BSMAS scores, suggesting impaired language performance in these subjects. According to our knowledge, evidence of altered language networks has not been provided in these conditions before. Only one study investigated verbal skills in PIU. Accordingly, Nie et al. (2017) reported significantly poorer language performance in severe problematic Internet users. They also claimed that it is impossible to decide whether the existing psychological-cognitive factors were the causes of PIU or if it works the opposite way. Our results indicate that there is a direct association between these addiction forms and language function independently of other cognitive domains. We assume that fewer personal social interactions, new communication forms, or the breakthrough of audio-visual technology may be the main reasons behind these associations (Kraut et al., 1998; Soleymani & Farahati, 2014; Venter, 2017).

It is not surprising that we observed negative associations between SABAS scores and FC in the executive control network and the left frontoparietal network, which are responsible for control and executive functions. Our findings support the hypothesis that impaired executive functions and weakened inhibition control play a crucial role in the pathogenesis and maintenance of addictions (Horvath et al., 2020; Lin et al., 2015; Sharifat et al., 2018; Wang et al., 2019; Wilmer et al., 2017).

There are some limitations that must be considered. The most significant is the lack of behavioral data regarding language performance. Although one previous study revealed impaired verbal fluency in PIU, without behavioral data, uncertainty surrounds the interpretation of our results. The cross-sectional nature of the study limits our ability to discriminate between cause and effect. It is impossible to determine whether alterations in FC lead to addictive behavior or vice versa. To get a clearer picture, longitudinal studies are needed. The generalization of our results may be weakened by the fact that most of the participants included in our study reported low or medium scores on the questionnaires. A new study in the future with addicted cohort and age-sex-education-matched control group would overcome this issue. Another limitation is the high intercorrelation amongst BSMAS and SABAS scores, that makes the interpretation of the related result difficult.

## Conclusion

Generally, smartphone and social media addictions lead to decreased FC in visual and language networks. Additionally, the executive control network and the frontoparietal network showed decreased within-network FC in people with high levels of smartphone addiction. These findings address the neural background of decreased verbal fluency performance reported previously in the literature. Moreover, we suggest that increased time spent with screen media devices has a negative effect on the functional organization of the visual networks. However, it is important to highlight that without well-established longitudinal studies, the causality should be interpreted carefully.

## Data Availability

The datasets generated during and/or analysed during the current study are available from the corresponding author on reasonable request.
